# Improving data quality across 3 sub-Saharan African countries using the Consolidated Framework for Implementation Research (CFIR): results from the African Health Initiative

**DOI:** 10.1186/s12913-017-2660-y

**Published:** 2017-12-21

**Authors:** Sarah Gimbel, Moses Mwanza, Marie Paul Nisingizwe, Cathy Michel, Lisa Hirschhorn, the AHI PHIT Partnership Collaborative, Ahmed Hingora, Dominic Mboya, Amon Exavery, Kassimu Tani, Fatuma Manzi, Senga Pemba, James Phillips, Almamy Malick Kante, Kate Ramsey, Colin Baynes, John Koku Awoonor-Williams, Ayaga Bawah, Belinda Afriyie Nimako, Nicholas Kanlisi, Elizabeth F. Jackson, Mallory C. Sheff, Pearl Kyei, Patrick O. Asuming, Adriana Biney, Roma Chilengi, Helen Ayles, Moses Mwanza, Cindy Chirwa, Jeffrey Stringer, Mary Mulenga, Dennis Musatwe, Masoso Chisala, Michael Lemba, Wilbroad Mutale, Peter Drobac, Felix Cyamatare Rwabukwisi, Lisa R. Hirschhorn, Agnes Binagwaho, Neil Gupta, Fulgence Nkikabahizi, Anatole Manzi, Jeanine Condo, Didi Bertrand Farmer, Bethany Hedt-Gauthier, Kenneth Sherr, Fatima Cuembelo, Catherine Michel, Sarah Gimbel, Bradley Wagenaar, Catherine Henley, Marina Kariaganis, João Luis Manuel, Manuel Napua, Alusio Pio

**Affiliations:** 1 School of Nursing, University of Washington, Magnuson Health Sciences Building, Box 357262, Seattle, WA 98195-7262 USA; 2 Department of Global Health, University of Washington, Seattle, WA USA; 3 Health Alliance International, Seattle, WA USA; 4 Centre of Infectious Diseases in Zambia, Lusaka, Zambia; 5 Partners in Health-Inshuti Mu Buzima, Kigali, Rwanda; 6 Health Alliance International, Beira, Mozambique; 7 Division of Global Health Equity, Brigham and Women’s Hospital, Boston, MA USA; 8 University of Global Health Equity, Kigali, Rwanda; 9 Feinberg School of Medicine, Northwestern University, Chicago, IL USA

**Keywords:** Data quality assessment, Quality improvement, Decision making, Health systems research, Health systems strengthening, Maternal and child health, Mozambique, Rwanda, Zambia

## Abstract

**Background:**

High-quality data are critical to inform, monitor and manage health programs. Over the seven-year African Health Initiative of the Doris Duke Charitable Foundation, three of the five Population Health Implementation and Training (PHIT) partnership projects in Mozambique, Rwanda, and Zambia introduced strategies to improve the quality and evaluation of routinely-collected data at the primary health care level, and stimulate its use in evidence-based decision-making. Using the Consolidated Framework for Implementation Research (CFIR) as a guide, this paper: 1) describes and categorizes data quality assessment and improvement activities of the projects, and 2) identifies core intervention components and implementation strategy adaptations introduced to improve data quality in each setting.

**Methods:**

The CFIR was adapted through a qualitative theme reduction process involving discussions with key informants from each project, who identified two domains and ten constructs most relevant to the study aim of describing and comparing each country’s data quality assessment approach and implementation process. Data were collected on each project’s data quality improvement strategies, activities implemented, and results via a semi-structured questionnaire with closed and open-ended items administered to health management information systems leads in each country, with complementary data abstraction from project reports.

**Results:**

Across the three projects, intervention components that aligned with user priorities and government systems were perceived to be relatively advantageous, and more readily adapted and adopted. Activities that both assessed and improved data quality (including data quality assessments, mentorship and supportive supervision, establishment and/or strengthening of electronic medical record systems), received higher ranking scores from respondents.

**Conclusion:**

Our findings suggest that, at a minimum, successful data quality improvement efforts should include routine audits linked to ongoing, on-the-job mentoring at the point of service. This pairing of interventions engages health workers in data collection, cleaning, and analysis of real-world data, and thus provides important skills building with on-site mentoring. The effect of these core components is strengthened by performance review meetings that unify multiple health system levels (provincial, district, facility, and community) to assess data quality, highlight areas of weakness, and plan improvements.

## Background

High-quality data are critical to inform, monitor and manage health programs [1]. Routine health management information systems (HMIS) encompassing national, subnational, facility and community levels are the primary data source for routine health planning and evaluation [2]. Access to comprehensive, accurate data to guide resource allocation and programmatic improvement efforts is increasingly important given: 1) continued high disease burden and resource constraints in many low- and middle-income countries (LMICs); 2) rapid implementation and scale-up of efficacious diagnostic, preventive and therapeutic interventions with significant personnel demands; and 3) plateauing of funding through global health initiatives [3].

In many resource-limited settings, accurate, high quality health data – defined as data that are consistently available, reliable across health system levels, and accurate when compared to population-level surveys – are often not available [4–6]. Poor quality routine data contributes to poor decision-making, inefficient resource allocation, loss of confidence in the health system, and may threaten the validity of impact evaluations [7]. Substantial investments have been made over the last decade to improve the quality and availability of essential health services [8]. Integrated monitoring and evaluation (M&E) systems to identify effective strategies may increase access to – and utilization of – these services [9]. However, considerable work is still needed to strengthen health information systems in many LMICs, including addressing challenges resulting from sometimes onerous donor reporting requirements. Donor-driven data requirements often result in duplicate reporting systems, burdening the limited numbers of health managers at health facility and district levels, which contributes to poor data quality [10].

Data quality assessment (DQA) approaches range from training program managers to audit data availability and reliability, to external audits of data quality [11]. Most data quality improvement efforts promoted by global health initiatives, bilateral donors, host countries, foundations and non-governmental organizations target disease-specific indicators, and are reliant upon continued external funding [12]. By not supporting data improvements across the broader primary health care system, these disease specific data quality assurance strategies undermine countries’ efforts to use high quality evidence for planning and resource allocation across programs, and the development of integrated and sustainable HMIS.

Over the seven-year African Health Initiative (AHI) of the Doris Duke Charitable Foundation, three of the five country Population Health Implementation and Training (PHIT) partnership projects supported by the initiative introduced context specific strategies to improve the quality of routinely collected data at the primary health care level, and stimulate the use of data for decision-making [13, 14].

In Mozambique a range of data quality assessment activities, including supportive supervision, training, dashboards, audits, etc., were employed. All 146 health facilities in the study province, Sofala, (pop = 1,500,000) received some form of DQA support. Annual data quality audits were carried out in a sample of 26 health facilities, including the largest health facility and two additional sites in each district and the regional quaternary-level facility. In Rwanda, the PHIT project targeted two districts, southern Kayonza and Kirehe (pop = 460,000) in eastern Rwanda, where 21 health centers and two district hospitals received targeted support to improve data quality and use. In Zambia, three rural districts in the Lusaka Province were targeted: Chongwe, Kafue and Luangwa (population over 300,000), where 42 health facilities were targeted for data quality investments.

These strategies reflected the heterogeneity of country contexts in Mozambique [3], Rwanda [15], and Zambia [16], and lessons learned from their implementation can contribute to the evidence-base of how to strengthen HMIS in LMICs to support data-driven decision making at all levels of the system. Using the Consolidated Framework for Implementation Research (CFIR) as a guide [17], this paper 1) describes and categorizes data quality assessment and improvement activities of PHIT partnership projects, and 2) identifies PHIT project core intervention components and implementation strategy adaptations to improve data quality.

## Methods

The CFIR is an evidence-based meta-framework drawing from psychology, sociology, organizational change, and other disciplines to provide a comprehensive, multidisciplinary taxonomy of constructs influencing implementation of complex interventions. The framework includes five major domains with associated constructs. The CFIR was adapted through a qualitative theme reduction process involving discussions with key informants from each PHIT project, who identified two domains and 10 constructs most relevant to the study aim of describing and comparing each country’s data quality assessment approach and implementation process [17]. These two domains are: 1) characteristics of the data quality improvement strategy (innovation source, evidence strength and quality, relative advantage, adaptability, complexity, cost); and 2) process used to implement the strategy (quality and extent of planning, engagement with key stakeholders, execution, reflection and evaluation).

Data were collected on each PHIT project’s data quality improvement strategies, activities implemented, and results – including adaptations over the course of the project – via a semi-structured questionnaire with closed and open-ended items administered to HMIS leads in each country initiative (*n* = 3), with complementary data abstraction from program reports. Findings were collated into the 10 identified CFIR constructs and presented to PHIT project implementation and HMIS leads in country-specific memos by the lead author to confirm accuracy.

A modified Delphi approach [18] with PHIT project leads was used to establish a convergence of opinion on core data quality intervention components and effective implementation strategy adaptations. This approach included administering a follow-up questionnaire to four HMIS leads (one each from Mozambique and Zambia, and two from Rwanda) via in-person interviews or one-on-one phone calls. After communicating individually with the lead author, each HMIS lead met with their country teams to confirm which implementation strategy adaptations were effective, to gain consensus on best practices to improve data quality, and to define core country-level activities. The HMIS leads in each country fed this information back to the lead author, who collated and synthesized findings into a cross-country memo. Cross-country core activity ranking included cross-checking country-specific rankings. Activities carried out and ranked essential across all three settings were ranked highest, followed by activities carried out and ranked essential across two countries. No activity was carried out and ranked as essential in only one country. This document, which included descriptions of effective implementation strategy adaptations, best practices and ranked data quality intervention activities, was shared with the HMIS leads to solicit feedback on accuracy and to resolve any disagreements or discrepancies in the findings. Finally in one setting, Rwanda, qualitative feedback was solicited from non-project respondents, namely beneficiary staff.

## Results

Here we describe PHIT project data quality improvement approaches in Mozambique, Rwanda and Zambia, guided by the 10 selected CFIR constructs (Table 1), followed by a description of core and adaptable strategy components as ranked by country teams. Detailed descriptions of each PHIT project’s health system strengthening interventions are summarized (Table 2); further descriptions have been previously published [3, 15, 16].

**Table 1:** Definitions of CFIR Constructs—Innovation and Process Domains

I. Innovation Characteristics	Definition
Innovation Source	*Perception of key stakeholders about whether the data quality improvement strategy/activities is externally or internally developed*
Evidence Strength and Quality	*Stakeholders’ perceptions of the quality and validity of evidence supporting the belief that the data quality improvement strategy/activities will have desired outcomes*
Relative Advantage	*Stakeholders’ perception of the advantage of implementing the data quality improvement strategy/activities* versus *an alternative solution*
Adaptability	*The degree to which the data quality improvement strategy/activities can be adapted, tailored, refined or reinvented to meet local needs*
Complexity	*Perceived difficulty of the data quality improvement strategy/activities, reflected by duration, scope, radicalness, disruptiveness, centrality and intricacy and number of steps required to implement*
Cost	*Costs of the data quality improvement strategy/activities and costs associated with implementing the innovation including investment, supply, and opportunity costs*
II. Implementation Process	Definition
Planning	*The degree to which a scheme or method of behavior and tasks for implementing the data quality improvement strategy/activities are developed in advance, and the quality of those schemes or methods*
Engaging (Opinion leaders, Formally appointed internal implementation leaders, Champions, External change agents, Key stakeholders, Innovation participants)	*Attracting and involving appropriate individuals in the implementation and use of the data quality improvement strategy/activities through a combined strategy of social marketing, education, role modeling, training and other similar activities*
Executing	*Carrying out or accomplishing the implementation of the data quality improvement strategy/activities according to plan*
Reflecting & Evaluating	*Quantitative and qualitative feedback about the progress and quality of implementation of the data quality improvement strategy/activities accompanied with regular personal and team debriefing about progress and experience*

**Table 2:** Partnership Data Quality Approaches

Mozambique	
Description of data quality improvement strategy *Introduction of simplified tools and strategies to strengthen the routine HMIS data system, with training and mentorship to district and facility managers provided to support the use of these strategies to improve health system performance.*	
Activity	Description
Data Quality Audits (DQA)	Annual DQAs are carried out in all districts in the intervention province of Sofala. Immediately after data collection, summary analyses are shared with district officials. Final written feedback is provided to all district and facility managers via a simplified, summary data quality ranking tool.
Data Dashboards	Quarterly development of data dashboards to simplify data visualizations to drive resource allocation & decision-making
Follow-up supportive supervision/ mentorship	Annual DQA results fed back to health facilities, districts and provinces and inform ongoing, targeted supportive supervision for sites with weaker clinical services, data quality and data utilization activities. Senior M&E mentor embedded within provincial health department.
Monitoring & Evaluation Training	Adaptation of existing MOH monitoring and evaluation training module, targeting primary health care strengthening.
	Quality improvement and operations research trainings with mentored support for subsequent applied research activities.
	Site-level trainings on HMIS functioning and use & basic Excel utilization at regular intervals over the LOP
District performance review and enhancement meetings (DPREM)	Meetings, targeting maternal child services, malaria and pharmacy, bring together health facility staff and district/provincial supervisors to review and analyze routine data.
Rwanda	
Description of data quality improvement strategy *Expand levels of routine data assessment (to include community level health data collection) as well as the frequency of audits in order to better integrate assessment into practice, which will translate into better use of data for decision-making in program management and evaluation.*	
Activity	Description
Enhanced Electronic Medical Records (EMR)	Electronic medical records are supported in the intervention districts to improve the quality of routinely collected data.
Data Quality Audits (DQA)	Quarterly DQAs facility reports versus HMIS data using patient registries, monthly reports and online HMIS data the project
	Monthly DQA in 2 districts between household registers and the community info system data
	Weekly data validation of IMB HIV EMR data
Community level lot quality assurance sampling (LQAS)	Community level data is assessed quarterly for concordance and completeness using LQAS methods where data is randomly sampled and five key indicators are compared with the database
Mentoring to enhance supervision in health centers (MESH) quality improvement efforts	LQAS methodology is used to assess the effect of supporting enhanced supervision and mentorship.
Assessment of HMIS using WHO data quality report card	Annual consistency and internal validity assessment
Data sharing and coordination meetings	Monthly data review meetings between project and district health staff
Zambia	
Description of data quality improvement strategy *Introduction of quality improvement teams across the project area who train facility and community-based health workers on filling of clinical forms, competency in following treatment protocols, overseeing data collection and ensuring quality of data entry*	
Activity	Description
Promoting completeness of clinical forms	Streamlining of clinical guides, including for case management of regularly seen presentations during patient visits, appropriate documentation carried out to improve clinician understanding of MOH-approved treatment protocols and tracking of stock outs of essential medicines and supplies
Expanded electronic medical record (EMR) system	Introduction of on-site, facility- and community-level electronic medical record system (EMR) in the target districts. This system automatically generates clinic, patient review, clinic performance, CHW performance (to track lost-to-follow-up patients), and HIS reports, using MOH data and shared with district level team to inform management decisions.
Community-based DQA component	Introduction of data quality audit system for community health information system
	Mentoring to develop and enhance good clinical skills and practices of MOH staff to improve key performance indicators related to clinical care quality
Continuous, on-site mentoring of MOH staff	Streamlining of clinical guides, including for case management of regularly seen presentations during patient visits, appropriate documentation carried out to improve clinician understanding of MOH-approved treatment protocols and tracking of stock outs of essential medicines and supplies
	Monthly, project and MOH staff assessed completeness of clinical forms, vital signs recorded, primary diagnosis made, case conclusion, accuracy of data entry by Clinic Supporters, successful referrals to the facility, and household surveys completed
Community outreach	Active data collection at household level, patient follow up and referral system carried out by community health workers, monitored through LQAS.

### Country approaches to improve data quality by CFIR constructs

#### Innovation characteristics

##### Innovation source

In all three PHIT projects, many data quality improvement activities were initially viewed as externally developed by academic entities within each project, though perceptions evolved as in-country members adapted existing data quality activities and introduced new activities suited to the implementation contexts.

Elements of the Mozambique data improvement strategy were seen as externally developed (data quality audits, data dashboards), while others were viewed as internally sourced (district performance review and enhancement meetings (DPREMs), and supportive supervision/mentoring and training). Ministry of Health (MOH) engagement and leadership in planning data quality assessments increased over the project period, which led to their increased use.

*“We used the audits as a learning activity so we got the district managers involved in going to facilities, and they got the preliminary results the same day so they were making decisions right away to improve.”* (Project lead, Mozambique)

MOH partners were less enthusiastic about dashboards – in spite of multiple adaptations – and these were eventually dropped.

*“We learned that the dashboards were too complicated and they were not taken up by the district managers, so we actually dropped this (dashboards).”* (Project lead, Mozambique)

In Zambia, both community-based DQA efforts and the broader quality improvement (QI) approach were viewed by MOH staff as externally conceptualized. Active engagement of Community Health Workers (CHWs) and facility staff in the community DQA design increased MOH ownership and strengthened links between CHWs and MOH staff. The introduction of externally developed QI mentorship teams was initially met with weak facility-level staff involvement, but intensified engagement with district supervisors (e.g. enlisting them as training and supervision mentors) improved participation of health facility personnel in QI work, including data quality tasks [19].

In Rwanda, respondents noted success in improving data quality led to countrywide adoption of specific project activities (such as audits), and broader routine data use.

*“Since the quality of data has been improving over time through these checks, these data are now used by researchers and investigators. These activities have increased awareness of data quality assessments, and led to increased data use not only at PHIT supported districts but also at national level where these assessments are now done routinely.”* (Project staff, Rwanda)

##### Evidence strength and quality

Initially, no background evidence was presented to stakeholders in the three countries to explain why data quality improvement approaches were needed. However, over the course of implementation, stakeholder perceptions in two of the three countries shifted to support data quality improvement strategies as data availability and consistency improved. In Mozambique, substantial HMIS improvements in data availability (84% to 99%) and consistency (54% to 87%) led to targeted resource allocation, including intensive training and supervision of weaker performing facilities and districts [20].

*“Now the (provincial health department) has added a mini data quality audit tool into the facility supervision guides, so each time the provincial or district supervisor visits a facility they assess concordance between registries and facility forms.”* (Project lead, Mozambique)

In Rwanda, investing in community HMIS improved linkages between facility and community health programs, as reliable, real-time data collected by CHWs informed health system planning.

*“The (CHW) data showed that there are children in the community who did not receive vaccines, we then went to the community to find the children and gave them vaccines.”* (MOH worker, Rwanda)

In Zambia, the MOH did not allocate personnel to participate in lot quality assurance sampling (LQAS), as perceived value was low – an example of how lack of information on evidence strength and quality may have contributed to suboptimal implementation.

*“The project had to collect all of the data for the LQAS, there was no support from the district health office as they didn’t see the point of it.”* (Project staff, Zambia)

##### Relative advantage

Prior to DQA initiation, routine HMIS’s in project countries were characterized as having poorly available and inconsistent data, which hindered their systematic use for planning and management (e.g. only 14% of MOH data were available in Zambia at baseline [21]; in other PHIT projects, data availability was higher (Mozambique 84% [20], Rwanda, 88%) [22]. However, initial data concordance was low in all countries [20–22]. As project implementation progressed and data quality improved, the perceived relative advantage of improvement strategies was recognized by health workers, MOH managers, Non-governmental organizations and donors, reinforcing and facilitating efforts. In Zambia, introduction of community DQA linked to facilities created bi-directional feedback mechanisms between facilities and communities, resulting in better mortality tracking, improved referral rates and community outreach.

*“Today we cannot send a report before we analyze it and double check its completeness and accuracy…the staff is paying much (more) attention on the data than they used to.”* (MOH, Rwanda)

*“There is a general feeling that Sofala province (project area) data were bad but have improved. Sofala is seen as managing routine data better than other provinces nationally.”* (Project lead, Mozambique)

*“…and it is a capacity building exercise on how to look at dashboards, on how to identify hot spots and all of those capacity building efforts we think go a long way towards those in leadership and management to actually use data on a regular basis to make decisions on their own programming and work planning.”* (Project staff, Rwanda)

##### Adaptability

Stakeholders reported that adapting improvement activities to the local context facilitated implementation. Iterative refinements were made based on MOH and community partner feedback, and best practices shared across PHIT projects at annual meetings, which increased data quality and its use for decision-making. Timing of feedback varied depending on the regularity of the data improvement activity. Facilities with electronic medical records (EMRs) were able to provide monthly and quarterly results to MOH; thus inconsistencies could be immediately addressed. In Mozambique, DQA activities were carried out annually, so refinements were carried out with less frequency.

In Rwanda, adaptations included increasing staff and resources for data system improvements, initiating meetings to discuss data with MOH partners, and expansion to include community HMIS. In Zambia, to improve continuity and effectiveness, supportive supervision evolved from a model led by the project QI team to a joint supervision model (conducted together with district MOH supervisors).

The presentation of DQA results in Mozambique was simplified to include an easy-to-read, color-coded (green/yellow/red) ranking of facilities by data quality over time, which were widely distributed to facilities and districts. To facilitate programmatic decision-making, trainings were expanded from statisticians to include general, disease-specific, and service-area managers. Periodic refresher trainings were added to address staff turnover.

##### Complexity

Perceived difficulty in implementing data quality improvements eased over time after adaptations were made or after repetition, with few exceptions that were discontinued. In Mozambique, frontline staff training was streamlined to include simple paper and Excel©-based graphing, and measures to encourage skills application were added to supervision checklists (e.g. % facilities with graphs in view). Five-day DPREMs were initially challenging, but as individuals participated in multiple cycles, their perceived complexity decreased. Conversely, data dashboards were viewed as time and labor intensive by MOH and project staff, and were discontinued.

In Zambia, key informants reported that the EMR facilitated tracking and reviewing patient records by clinicians at study facilities. Likewise, engaging facility staff in on-site reviews enabled prompt decision-making in areas requiring improvement. Although CHWs were able to conduct the majority of household surveys (80%), mobile data collection was difficult due to frequent service disruptions and no back-up data system, and was not adopted by the MOH.

Challenges with EMR complexity in Rwanda were countered by recruiting a technical expert who worked with project and MOH staff to transfer skills. Automated quality checks were integrated into the EMR, and additional resources invested to augment clinician EMR training.

*“We provided more training for clinicians about EMR. Clinicians were trained on using EMR, so that they can enter the program and run the report any time they feel they need it.”* (Project staff, Rwanda)

##### Cost

PHIT project teams noted substantial costs with data quality improvement activities. Staff in Mozambique noted the high DQA and DPREM cost, and though work was planned and managed by the MOH, funding came through external sources. Although frequency and intensity of exercises should decrease over time as data quality improves, resources will still be required to maintain these activities. Some districts have begun to identify their own funding sources to maintain activities, and new donors have expressed interest in continuing support. In Rwanda, to continue DQAs, support for hiring data coordinators was included within block grant financial support from the PHIT project (managed through the state).

In Zambia, financial costs were important for sustainability. Mobile phone data uploaded to the EMR by CHWs was human resource intensive and financially unsustainable, while the (low-cost) use of clinical decision-making forms will be independently continued by the MOH. Additive human resource support for EMR capacity building within the MOH was provided by the Rwandan PHIT project but not by the Zambia project, which ultimately limited its ability to systematically disseminate findings.

#### Implementation process

##### Planning

Data quality improvements in Mozambique were jointly designed with MOH stakeholders. The district focus also aligned with ongoing decentralization processes that increase responsibilities for district managers in activity monitoring and resource allocation. DQA piloting to assess feasibility and effectiveness in Mozambique and Rwanda supported their subsequent broader use [22, 23].

In Zambia, MOH engagement in planning and implementing electronic medical records, clinical protocols, and clinical forms for the community, facility and district levels, facilitated their incorporation into routine HMIS. A baseline capacity assessment prior to community DQA implementation [24] was essential in establishing feasibility, and supported mobile-phone based data collection [25]. Zambia reported that the joint engagement of district and facility health staff in all aspects of data quality work increased ownership and commitment to improving performance, through the development of networks which helped diffuse best practices and performance improvements.

##### Engaging

In Mozambique, sub-national supervisors championed data quality improvement through DPREM leadership, which iteratively facilitated ownership and skills development. Likewise, when Rwandan MOH managers participated in HMIS-linked planning meetings to allocate resources, the perceived value of data quality improvement activities increased.

In Zambia, involvement of stakeholders in improving data quality increased over the project, but challenges with health worker motivation persisted, possibly attenuating data improvement effects [26]. Integration of mentoring and supervision tools into monthly district health supervision and data quality audits proved feasible, but joint LQAS proved unsuccessful.

##### Executing

In Mozambique, DQA implementation and dissemination was conducted as planned, and encouraged healthy data quality competition across facilities. Linking DQA results with targeted supervision was implemented across all three PHIT projects, which helped ensure data were used for service improvements.

In Rwanda, data assessments and mentored identification of data quality gaps helped integrate data quality activities into routine health worker practice, and further helped articulate clearer feedback loops between health workers and managers. In all countries, facility-level implementation increased with greater ownership by facility leadership and data managers. Finally, the use of data for results-based health center financing and district collaboratives increased the value of work to ensure data quality [27, 28].

##### Reflecting and evaluating

In Zambia, engagement between study QI teams and district management staff was reinforced in the first year of implementation through facility-level meetings to clarify roles/responsibilities of district and facility staff. Reinforcement of technical capacity, management coordination and communication was critical in all PHIT projects to facilitate integration of study activities into routine practice.

Rwanda’s reflection and evaluation period focused on team-building. Clinicians and EMR data officers, hired through support of the PHIT project, were brought together to jointly analyze data and address inconsistencies, and review findings during dissemination which was essential for skill development and sustained engagement. District-level data sharing meetings reinforced initial engagement and solidified buy-in.

*“Before…the staff were not curious to explore the data. The staff did not give much value to data, but today, everyone is concerned, and we work in collaboration to improve the data quality.”* (Rwanda Project Staff)

Data quality improvement in Mozambique included multiple feedback loops to facility, district, and provincial managers, including dissemination of annual DQA results, participation in DPREMs, and supportive supervision visits. DPREMs proved particularly effective, as they provided space for provincial, district and facility staff to present and review secular trends in outputs and coverage estimates across a range of indicators, and jointly develop collaborative action plans to address performance gaps.

### Core and adaptable components of the data quality improvement approach

**Fig. 1:**
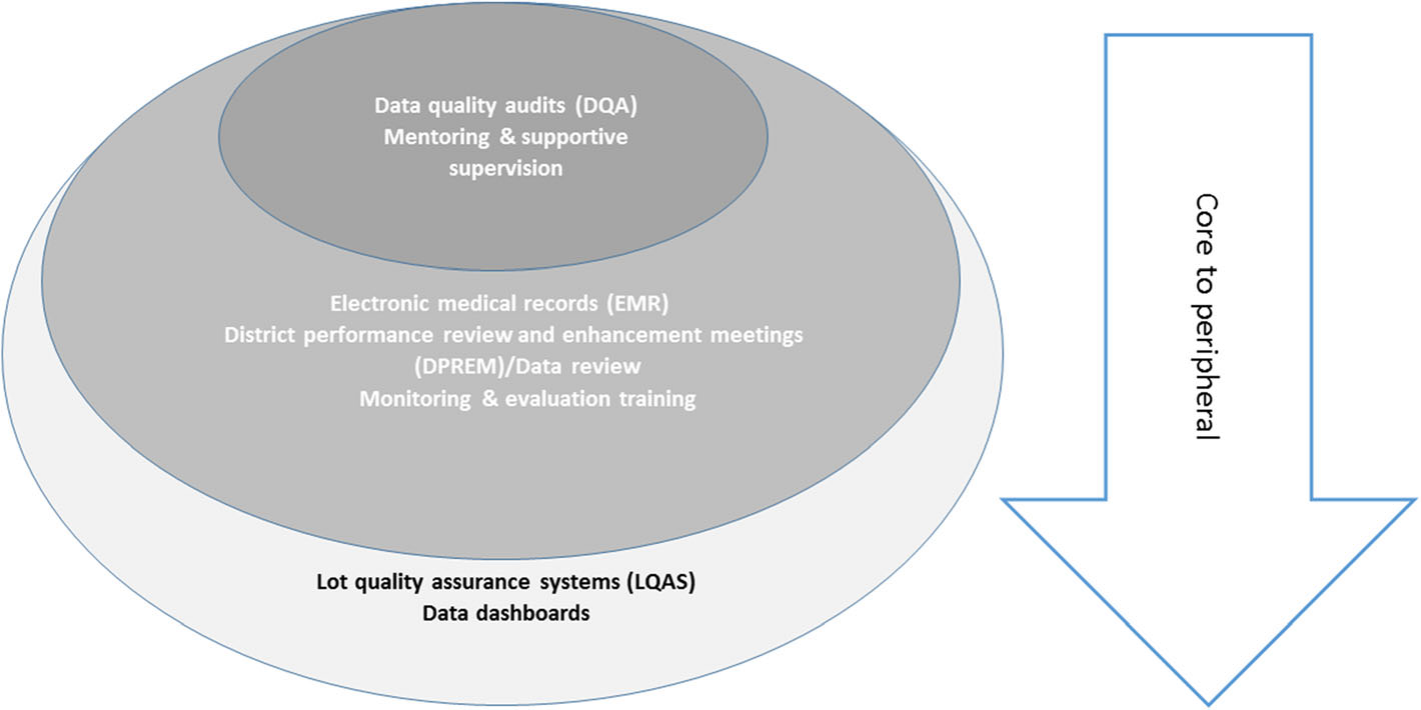
Core activities ranked by importance across sites

Across the three PHIT projects, routine DQA and engagement through mentoring/supportive supervision were identified as core intervention components. However, as projects were introduced in different contexts with unique data quality challenges and priorities, implemented activities were classified along a continuum from core, (or essential to improving data quality) to peripheral, or (non-essential to improving data quality), reflecting the degree of applicability across the three settings (see Fig. 1). Intervention activities targeted different health system levels, and in some settings, the community-level (Table 2).

**Fig. 2:**
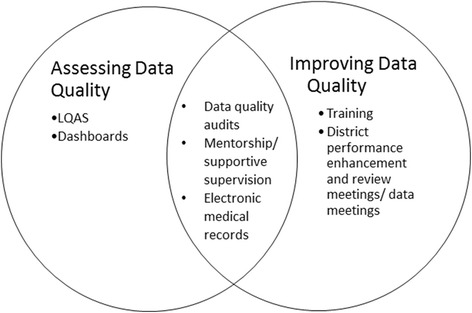
Categorization of data quality activities

Activities that both assessed and improved data quality (including DQAs, mentorship/supportive supervision targeting data quality improvement, and establishment and/or strengthening of EMR system quality), received higher ranking scores from respondents (see Fig. 2). Across PHIT projects, these activities were also identified as effectively engaging MOH partners to varying degrees in the design, data collection and analysis.

Given that insufficient levels and training of health personnel are a fundamental health system constraint across the PHIT project countries, the focus on mentorship and supportive supervision to improve data quality is appropriate. Data dashboards, designed to give a “snapshot” of health service data to district managers to inform decision-making, were introduced in each project, but only continued in one, Rwanda, with substantial external technical support and financial resources.

Two projects implemented each of the following activities – meetings to review data quality and use for identifying service gaps and targeting solutions; training for institutional and CHWs in data management, planning, and M&E; and LQAS to determine concordance of service output measures from community to facility levels. Respondents reported activities directly linked to other data quality activities were seen as more central to the broader health information system strategy and the overall PHIT project strategy, while those whose evidence strength and fit with ongoing data quality improvement activities were not effectively conveyed to MOH stakeholders, were not as helpful. Thus, the engagement of supportive supervision and mentorship targeting data quality, which was informed by DQA results and subsequently discussed in data review meetings, were viewed more favorably than LQAS and data dashboards which were carried out independent of other data quality activities and were seen as externally pushed by PHIT projects.

## Discussion

The experience of three diverse PHIT projects contributes to existing evidence that district, health facility and community-based data quality improvement activities are an essential element of health system strengthening, helping to ensure that reliable data is available for prompt decision-making [6, 23, 29, 30]. These strategies must reflect the heterogeneity of country contexts, including adaptable approaches for HMIS strengthening.

Through the lens of the CFIR, commonalities in effective data quality strengthening were identified despite the diversity of intervention models and settings. HMIS quality must be routinely reviewed, including cross-checks between facility/district/provincial levels. This process is strengthened when roles and responsibilities across stakeholders are clarified. Articulated feedback loops between collection, management and policy help ensure results are shared in a timely manner with stakeholders responsible for program development and service delivery. EMRs produced timely, high quality data which supported data-driven decision making and improvements in clinical care, as data accessibility facilitated routine validation. Expanding HMIS quality audits to monitor community-level health provision, when integrated into existing management systems, strengthened communication and collaboration between communities and facilities.

Engaging managers from facility, district, and provincial levels in data quality improvement design and adaptation, and subsequent data collection and analysis efforts, was fundamental to their sustainability, which is consistent with previous findings [10, 30, 31]. Stakeholder inclusion from multiple health system levels encourages cross collaboration and bolsters data-driven decision-making. For example, in Zambia, reinforcing district managers’ engagement led to the PHIT project’s design being integrated into the HMIS.

Our review found that linking data quality improvement approaches to one another reinforces them within a broader data systems strengthening framework. In Mozambique, DQA result dissemination was integrated into management strengthening and included targeting facilities for training and supervision. This integration unified multiple health system levels together to review facility level performance, building skills for facility-level managers in data management and interpretation, which reinforced participation of facility and district managers in subsequent DQAs.

## Conclusion

Complete, reliable and timely information is central to effective decision-making – which links across all health system building blocks (improved leadership/governance, healthcare financing, health workforce, medical products/technologies, information and research, service delivery). HMISs ideally serve multiple users across all levels of a health system with reliable information on which to base decisions – including problem identification, resource allocation, and program and policy development [32]. This article applies the CFIR to describe and frame discussion of the heterogeneous experiences implementing data quality improvement strategies across three sub-Saharan African countries involved in a health systems strengthening initiative, providing useful guidance to others improving health information systems in resource-constrained environments. Our findings suggest that, at a minimum, successful data quality improvement efforts should include routine audits linked to ongoing, on-the-job mentoring at the point of service. This pairing of interventions engages health workers in data collection, cleaning, analysis of real-world data, and thus provides important skills building with on-site mentoring. The effect of these core components is strengthened by performance review meetings that unify multiple health system levels (provincial, district, facility, and community) to assess data quality, highlight areas of weakness, and plan improvements. Other studies have demonstrated that with adequate financial and human resources, implementation of EMR systems can accelerate and systematize data feedback loops to core stakeholders, such as is the case in Rwanda [33]. In the three PHIT projects involved in this study, intervention components that aligned with user priorities and government systems were perceived to be relatively advantageous, and more readily adapted and adopted, which will facilitate efforts to sustain and spread data quality improvements.

## Abbreviations

AHI: African Health Initiative
CFIR: Consolidated Framework for Implementation Research
CHWs: Community Health Workers
DDCF: Doris Duke Charitable Foundation
DPREM: District Performance Review and Enhancement Meeting
DQA: Data Quality Audit
EMR: Electronic Medical Records
HMIS: Health Management Information System
LMICs: Low- and Middle Income Countries
LQAS: Lot Quality Assurance Sampling
M&E: Monitoring and Evaluation
MOH: Ministry of Health
PHIT: Population Health Implementation and Training partnerships
QI: Quality Improvement
